# Creating outbred and inbred populations in haplodiploids to measure adaptive responses in the laboratory

**DOI:** 10.1002/ece3.6454

**Published:** 2020-07-07

**Authors:** Diogo P. Godinho, Miguel A. Cruz, Maud Charlery de la Masselière, Jéssica Teodoro‐Paulo, Cátia Eira, Inês Fragata, Leonor R. Rodrigues, Flore Zélé, Sara Magalhães

**Affiliations:** ^1^ Centre for Ecology, Evolution and Environmental Changes – cE3c Faculdade de Ciências da Universidade de Lisboa Lisboa Portugal

**Keywords:** biological resources, experimental evolution, laboratory studies, quantitative genetics, spider mites

## Abstract

Laboratory studies are often criticized for not being representative of processes occurring in natural populations. One reason for this is the fact that laboratory populations generally do not capture enough of the genetic variation of natural populations. This can be mitigated by mixing the genetic background of several field populations when creating laboratory populations. From these outbred populations, it is possible to generate inbred lines, thereby freezing and partitioning part of their variability, allowing each genotype to be characterized independently. Many studies addressing adaptation of organisms to their environment, such as those involving quantitative genetics or experimental evolution, rely on inbred or outbred populations, but the methodology underlying the generation of such biological resources is usually not explicitly documented. Here, we developed different procedures to circumvent common pitfalls of laboratory studies, and illustrate their application using two haplodiploid species, the spider mites *Tetranychus urticae* and *Tetranychus evansi*. First, we present a method that increases the chance of capturing high amounts of variability when creating outbred populations, by performing controlled crosses between individuals from different field‐collected populations. Second, we depict the creation of inbred lines derived from such outbred populations, by performing several generations of sib‐mating. Third, we outline an experimental evolution protocol that allows the maintenance of a constant population size at the beginning of each generation, thereby preventing bottlenecks and diminishing extinction risks. Finally, we discuss the advantages of these procedures and emphasize that sharing such biological resources and combining them with available genetic tools will allow consistent and comparable studies that greatly contribute to our understanding of ecological and evolutionary processes.

## Graphical abstract

Here, we detail methodologies to create outbred and inbred populations for laboratory studies in haplodiploids. Additionally, we provide an experimental evolution protocol, which allows reducing the risks of losing experimental populations. Finally, we outline the advantages of such methodologies and we emphasize that combining these biological resources with available genetic tools will allow consistent and comparable studies among laboratories using the same organisms.

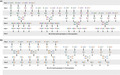

## 1 INTRODUCTION

Understanding the processes that shape individual traits and ecological processes in natural populations is arguably the ultimate aim of evolutionary ecology. This can be achieved by studying populations in their natural environment (Arnold, 1983). However, this approach suffers from the difficulty in controlling several environmental variables simultaneously (Lauder, Leroi, & Rose, 1993). Laboratory studies, in contrast, while allowing for controlled variables, are often criticized for not being representative of the processes occurring in natural populations (Aguilar, Dong, Warr, & Dimopoulos, 2005; Calisi & Bentley, 2009; Melvin & Houlahan, 2012). This is partly because it is not possible to recreate the complexity of the natural environment in the laboratory (Calisi & Bentley, 2009; Carpenter, 1996). Another reason is that laboratory populations often do not harbor sufficient variability to produce representative responses. Indeed, some studies have shown that laboratory populations have lower genetic variability than natural populations (Bian, Gao, Lamberton, & Lu, 2015; Norris, Shurtleff, Touré, & Lanzaro, 2001; Stohler, Curtis, & Minchella, 2004), possibly due to bottlenecks during the establishment and maintenance of the population, or to the long‐term adaptation to the same environment, that is, the laboratory conditions (Aguilar et al., 2005; Matos, Rose, Pité, Rego, & Avelar, 2000; Santos et al., 2012; Stohler et al., 2004). However, the lack of representativity of laboratory populations may also be related with the origin and the procedures involved in the creation of such populations (Berthier et al., 2010). Natural populations of the same species may be genetically differentiated and/or harbor different genetic compositions, shaped by different geographic and environmental factors (Aguilar et al., 2005; Bian et al., 2015; Langley et al., 2012; Nunes, Neumeier, & Schlotterer, 2008). Thus, laboratory populations founded by individuals collected from a single‐field population may not produce representative responses, even if the sample size is enough for the sample to be representative of that population.

To ensure that data obtained in the laboratory reflect the range of possible responses found in natural populations of the species under study, the ancestral population should reflect the variability found in the field (Faria & Sucena, 2017; MacDonald & Long, 2004; Nunes et al., 2008). Several studies have used laboratory populations founded by a large number of individuals collected in the field and maintained at high numbers in the laboratory (e.g., Magalhães, Fayard, Janssen, Carbonell, & Olivieri, 2007; Martins, Faria, Teixeira, Magalhães, & Sucena, 2013; Mery & Kawecki, 2002; Teotónio, Chelo, Bradić, Rose, & Long, 2009). However, this method falls short of accounting for potential geographic variation in trait values across populations. To ensure this, some authors have produced outbred populations by merging clones, inbred lines (King et al., 2012; Kover et al., 2009; Zbinden, Haag, & Ebert, 2008), or field populations (Fricke & Arnqvist, 2007; Tucic, Milanovic, & Mikuljanac, 1995) collected at different locations. This option increases the chance of obtaining a population containing genotypes from different environments, thus potentially representing different subsets of the genetic variability of a species. Yet, this procedure does not preclude the possibility that one (or a set of) genotype(s) from a particular environment is overrepresented in the final population. To circumvent this caveat, in sexual organisms, it is desirable to create an outbred population with an equitable representation of the genotypes present in several field populations, which can be achieved by performing controlled crosses between individuals of different populations.

Using outbred populations not only increases the representativity of the observed responses but also the robustness of comparisons between studies performed in different laboratories, if these populations are shared (Churchill, Gatti, Munger, & Svenson, 2012). Thus, with the exception of studies on local adaptation, such as common garden and reciprocal transfers experiments (Blanquart, Kaltz, Nuismer, & Gandon, 2013; Kawecki & Ebert, 2004), and of other studies aiming at comparing populations, most types of laboratory studies may benefit from using outbred populations. In particular, understanding the process of adaptation to specific selective pressures, in controlled laboratory conditions, with experimental evolution and quantitative genetic methodologies, requires the usage of populations with large amounts of variability (Kawecki et al., 2012; Svenson et al., 2012).

Experimental evolution follows adaptation of populations exposed to specific selection pressures in real time (Gibbs, 1999; Kawecki et al., 2012). Hence, it allows measuring the process of adaptation itself instead of inferring it based on observed patterns, and to infer causality. This method consists in deriving populations from a common ancestral and exposing them to specific controlled environments during several generations, which enables (a) knowledge of the ancestral state of populations (i.e., the ancestral population from which all others were derived), (b) the possibility to define and control the environments that populations are exposed to, and (c) replication at the population level (Magalhães & Matos, 2012). The explanatory power of experimental evolution can be used to unravel how populations adapt to environmental changes, to the presence of antagonists or to different population structures (Kawecki et al., 2012; Macke, Magalhães, Bach, & Olivieri, 2011; Rodrigues, Duncan, Clemente, Moya‐Laraño, & Magalhães, 2016; Zélé, Magalhães, Kéfi, & Duncan, 2018). Additionally, this method can be used to measure convergent evolution of different populations to a common environment (e.g., the laboratory; Fragata et al., 2014; Simões et al., 2008). In any case, adaptation of nonmicrobial organisms to rapid environmental changes relies mostly on the standing genetic variation present in a population, rather than on the arrival of new mutations (Barrett & Schluter, 2008; Hermisson & Pennings, 2005; Sousa et al., 2019). Thus, for the establishment of experimental evolution populations, it is crucial to generate and maintain populations with large genetic variability in the laboratory, being the above‐mentioned outbred populations an excellent tool for that purpose. Moreover, some populations may crash during the evolution process. Therefore, it is useful to design methods that maximize the prevention of such events.

Quantitative genetics uses several designs to evaluate the genetic versus environmental contribution to a particular phenotype (Falconer & Mackay, 1996). In such studies, it is important that the population used to infer these contributions is sufficiently variable. Some designs rely on a panel of inbred lines, which allows identifying any quantitative trait loci involved in one phenotype (Mackay, 2004). To ensure that this panel is composed of different genotypes, it is important to derive it from a highly outbred population (e.g., King et al., 2012; Mackay et al., 2012). Such panel can then be used to measure the broad‐sense heritability of a given trait, as well as genetic correlations between traits (e.g., Travers, Garcia‐Gonzalez, & Simmons, 2015; Howick & Lazzaro, 2017; Wang, Lu, & Leger, 2017; Lafuente, Duneau, & Beldade, 2018; Everman, McNeil, Hackett, Bain, & Macdonald, 2019. Although the most famous and complete panels are found in *Drosophila* (DGRP—Mackay et al., 2012; DSPR—King et al., 2012; GDL—Grenier et al., 2015), this resource has also been used in plants (Kover et al., 2009; Wills et al., 2013) and other animals (Table 1). Outbred populations themselves may also be useful in quantitative genetic designs (Solberg Woods, 2014; Svenson et al., 2012). In contrast with inbred lines, where each line represents a fixed allelic combination, individuals from outbred populations are maintained in randomized recombinant crossings. Therefore, from an outbred population one can retrieve a much higher amount of allelic combinations, allowing a fine mapping of complex phenotypes (Solberg Woods, 2014; Svenson et al., 2012).

**TABLE 1:** Inbred line panels in different animal species

Organism	Characteristics	# of lines	Reference
*Drosophila melanogaster*	“DGRP” founded from 1 outbred population (1,500 mated females)	192/205	Mackay et al. (2012); Mackay and Huang (2018)
*Drosophila melanogaster*	“DSPR” founded from 2 outbred populations, created from 15 isolines, and recombined for 50 generations, followed by 25 generations of inbreeding	1,600	King et al. (2012)
*Drosophila melanogaster*	“GDL” founded from 5 populations coming from different geographic regions; inbred for 12 generations	84	Greenberg, Hackett, Harshman, and Clark (2010); Grenier et al. (2015)
*Drosophila simulans*	Founded from mated females collected from a single population; 15 generations of sib‐mating	170	Signor et al. (2018)
*Drosophila serrata*	Founded from mated females collected from a single population; 17–20 generations of sib‐mating	110	Reddiex, Allen, and Chenoweth (2018)
*Mus musculus*	“RIX” founded from 8 laboratory strains, combined during 3 generations, and then inbred during 20 generations	69	Churchill et al. (2004); Srivastava et al. (2017)
*Mus musculus*	“BXD ARI” founded from 2 laboratory strains (after 9–14 generations of intercrossing, followed by at least 14 generations of inbreeding	46	Peirce, Lu, Gu, Silver, and Williams (2004)
*Caenorhabditis elegans*	Founded from 2 wild‐type strains; 15 generations of inbreeding	73	van Swinderen et al. (1997)
*Caenorhabditis elegans*	Founded from 8 parental lines each initially crossed with males of one different line; 3–5 generations of backcrossing, followed by 10 generations of selfing	90	Doroszuk, Snoek, Fradin, Riksen, and Kammenga (2009)
*Caenorhabditis elegans*	12 lines from 2 hybrid populations (6 from each); 6 from 11 generations of selfing and 6 from 22 generations of sib‐mating	12	Teotónio, Carvalho, Manoel, Roque, and Chelo (2012)
*Caenorhabditis elegans*	359 genotypes from reciprocal crosses between 2 parental lines; 3 generations of backcrossing, followed by 10 generations of controlled matings between hybrids	359	Andersen et al. (2015)
*Caenorhabditis elegans*	Founded from 2 lines; 10 generations of selfing	120	Frézal, Demoinet, Braendle, Miska, and Félix (2018)
*Callosobruchus maculatus*	Founded from 215 mated females; 10 generations of inbreeding	~ 86 (40% of 215)	Bilde, Maklakov, Meisner, la Guardia, and Friberg (2009)

Note

# of lines—number of lines available.

Here, we describe the creation of the above‐mentioned biological tools, outbred populations and inbred lines, using protocols focused on haplodiploid systems. The creation of hybrid populations using controlled crosses in haplodiploids has an extra layer of complexity as compared to diploid species. This is because in these systems, females stem from fertilized eggs whereas haploid males stem from unfertilized eggs. Thus crosses between different genotypes/ populations only generate hybrid diploid females, and hybrid males can only stem from unfertilized eggs of these hybrid females. As a case study, we describe the creation of outbred populations for two species of haplodiploid spider mites. This was done by performing controlled single crosses between individuals of different populations, within each species, in round‐robin and matched crosses designs. From one of these outbred populations, we also created inbred lines through 15 generations of sib‐mating, for which we also present a method to calculate the coefficient of inbreeding through time, adapted to haplodiploid species, as well as a method to calculate the probability of having a fully inbred line. Finally, we provide a general description of an experimental evolution protocol, which includes a backup for each experimental population that can be used to replenish the population when needed, maintaining a constant population size at each transfer and minimizing the risk of extinction of such populations. With this work, we aim to provide the community with protocols that can be easily applied, not only to this, but to other systems.

## 2 COLLECTION OF FIELD POPULATIONS

**FIGURE 1:**
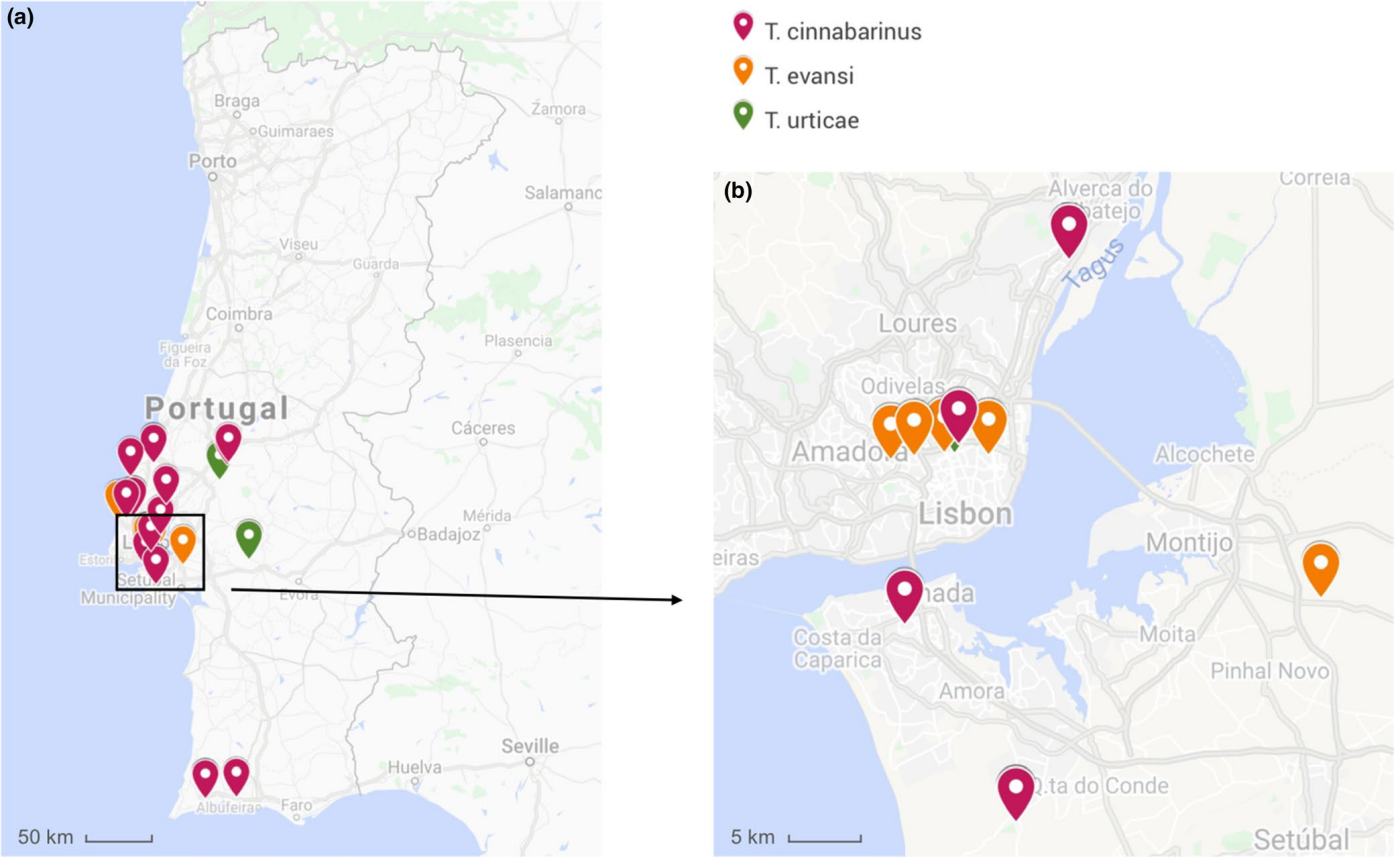
Map of the field sampling of spider mites. (a) Total of 24 sites visited in Portugal, (b) detailed Lisbon geographic region. This map was adapted from Google Maps (Google n.d. retrieved from https://www.google.com/maps/d/viewer?mid=1v4‐9f9Rgmc2HZOfI9TEeoMJB_yS9ZYVu&hl=en&usp=sharing)

In order to maximize the representativity of responses observed in laboratory studies, field populations used to create outbred populations should be sampled at different locations. Here, we used spider mites (Acari: Tetranichidae), which are haplodiploid pests widespread in many agricultural crops (Migeon, Nouguier, & Dorkeld, 2010). Given their small size and life‐cycle characteristics, these species are easily reared and maintained in high numbers in the laboratory. We surveyed several tomato (*Solanum lycopersicum*) fields and greenhouses in Portugal for the presence of Tetranychid mites between May and October 2017 (Figure 1). Each location was sampled during ca. 1 hr. Tomato leaves infested with spider mites were collected and kept in a closed plastic box. If the tomato plants were free of spider mites, neighboring plants from other species were also screened. All collected spider mite populations were established in the laboratory by transferring adult females (*N* = 32–463, depending on the density of spider mites found on a given location; Table 2) to a rearing cage containing tomato leaves (variety Moneymaker). Since arrival to the laboratory the populations were maintained under controlled conditions (23.5 ± 2°C, 60% RH, 16/8 hr L/D) for a few generations (3–6), populations were left untouched to promote laboratory adaptation and foster an increase in population size (>300 adult females). Subsequently, each population was identified at the species level by performing a multiplex PCR on a pool of 50–100 spider mites (Zélé, Santos, et al., 2018, detailed in Appendix S1). A total of 27 populations were collected in 24 different locations (Table 2). Sixteen of those were identified as *Tetranychus cinnabarinus* (also referred to as the red form of *Tetranychus urticae*; Auger, Migeon, Ueckermann, Tiedt, & Navajas Navarro, 2013), 4 as *T. urticae* (green form), and 7 as *Tetranychus evansi*. In 8 locations, there were no spider mites infesting tomato plants, but on 4 of those, spider mite populations were found on neighboring plants (Table 2).

**TABLE 2:** Geographic locations visited to sample spider mites

Location	Date	Host plant	# females	Population name	Species	Coordinates
Biomimos, Almada	30.05.2017	*Solanum lycopersicum* ^a^	0	n.a.	n.a.	38.657101, −9.183984
		Unknown	38	OB	*Tetranychus cinnabarinus*	
Quinta dos medronheiros, Sesimbra	30.05.2017	*Solanum lycopersicum* ^a^	0	n.a.	n.a.	38.543333, −9.101944
		*Phaseoulus lunatos*	54	MFV	*Tetranychus cinnabarinus*	
		*Solanum muricatum*	118	MPE	*Tetranychus cinnabarinus*	
Herdade do Freixo do Meio, Montemor‐o‐Novo	28.06.2017	*Solanum lycopersicum* ^a^	72	HFM (H)	*Tetranychus urticae*	38.703667, −8.325385
Quinta Vidigal, Montemor‐o‐Novo	28.06.2017	*Solanum lycopersicum* ^a^	146	MON (G)	*Tetranychus urticae*	38.706887, −8.327368
Chamusca	03.07.2017	*Solanum lycopersicum* ^a^	158	CHA1	*Tetranychus cinnabarinus*	39.317635, −8.506450
		*Solanum lycopersicum* ^a^	0	n.a.	n.a.	39.312217, −8.516502
		*Solanum lycopersicum* ^a^	0	n.a.	n.a.	39.330084, −8.492160
		*Solanum lycopersicum* ^a^	38	CHA2	*Tetranychus cinnabarinus*	39.333608, −8.498716
Agrial, Alpiarça	03.07.2017	*Solanum lycopersicum* ^a^	87	ALP (E)	*Tetranychus urticae*	39.221486, −8.572960
Quinta do Montalto, Ourém	04.07.2017	*Solanum lycopersicum* ^a^	0	n.a.	n.a.	39.698069, −8.598858
Gracieira, Óbidos	04.07.2017	*Solanum lycopersicum* ^a^	89	OTO	*Tetranychus cinnabarinus*	39.331097, −9.121093
		*Phaseoulus vulgaris*	300	OBE	*Tetranychus cinnabarinus*	
Campo Grande, Lisboa	11.07.2017	*Solanum nigrum*	320	CG	*Tetranychus evansi*	38.756088, −9.154691
Parque hortícola Aquilino Ribeiro Machado, Lisboa	11.07.2017	*Solanum lycopersicum* ^a^	207	LNEC	*Tetranychus cinnabarinus*	38.760753, −9.144024
Quinta da Granja, Lisboa	11.07.2017	*Solanum lycopersicum* ^a^	181	QG (C)	*Tetranychus evansi*	38.752069, −9.194166
Parque da Boa Saúde, Lisboa	11.07.2017	*Solanum lycopersicum* ^a^	153	PBS (B)	*Tetranychus evansi*	38.753958, −9.177114
Póvoa de Santa Iria	11.07.2017	*Solanum lycopersicum* ^a^	65	PSI	*Tetranychus cinnabarinus*	38.866650, −9.062553
Alenquer	29.07.2017	*Cucumis sativus*	32	ALE	*Tetranychus cinnabarinus*	39.063222, −9.018111
Hortas comunitárias do Vale de Chelas, Lisboa	10.08.2017	*Solanum melongena*	76	VC	*Tetranychus evansi*	38.754530, −9.122054
		*Solanum lycopersicum* ^a^	0	n.a.	n.a.	
Ericeira	16.08.2017	Unknown	94	ER	*Tetranychus evansi*	38.966135, −9.417877
Quinta das 6 Marias, Lagos	10.08.2017	*Solanum lycopersicum* ^a^	400	6M1 (A)	*Tetranychus evansi*	37.136506, −8.690885
		*Solanum lycopersicum* ^a^	93	6M2	*Tetranychus cinnabarinus*	
Quinta da Pedra Branca (Gradil)	14.08.2017	*Solanum lycopersicum* ^a^	74	GT	*Tetranychus cinnabarinus*	38.990052, −9.292190
		*Carica papaya*	100	GP	*Tetranychus cinnabarinus*	
Biobrotar, Mafra	19.09.2017	*Solanum lycopersicum* ^a^	0	n.a.	n.a.	38.975686, −9.350661
		Unknown	72	BB	*Tetranychus cinnabarinus*	
Biofrade, Lourinhã	22.09.2017	*Solanum lycopersicum* ^a^	52	BF	*Tetranychus cinnabarinus*	39.244005, −9.312744
Casa da Caldeira, Rio Maior	22.09.2017	*Solanum lycopersicum* ^a^	0	n.a.	n.a.	39.343618, −8.797132
Horticilha, Alcochete	13.10.2017	*Solanum lycopersicum* ^a^	317	VIT (D)	*Tetranychus evansi*	38.672328, −8.876891
Agrolimoa, Lagoa	13.10.2017	*Solanum lycopersicum* ^a^	463	LIM	*Tetranychus cinnabarinus*	37.147162, −8.432982
Alvalade, Lisboa	13.10.2017	*Solanum lycopersicum* ^a^	300	DEF (F)	*Tetranychus urticae*	38.755673, −9.147124

Note

For each location, the table includes the coordinates, the date and the host plants examined, as well as the number of females (# females) and their species when a population was found. Letters (A to H) correspond to the populations used in the creation of the outbred populations.

Abbreviation: n.a., nonapplicable.

^a^ Tomato.

## 3 CHARACTERIZATION OF FIELD POPULATIONS

In several organisms, different features of the field‐collected populations can lead to reproductive incompatibilities between different populations/genotypes and may hamper the maintenance of variability along the creation of outbred populations. Identifying the source of such incompatibilities, and excluding or avoiding them, is thus a prerequisite to the successful creation of outbred populations.

In many arthropod species, including spider mites, the presence of maternally inherited bacterial endosymbionts may hamper the viability of offspring from interpopulation crosses (Duron et al., 2008; Engelstädter & Hurst, 2009; Telschow, Hammerstein, & Werren, 2002). Therefore, we assessed infection by three of the most common reproductive manipulators found in arthropods (Weinert, Araujo‐Jnr, Ahmed, & Welch, 2015; including spider mites, e.g., Zélé, Santos, et al., 2018), namely, *Wolbachia*, *Cardinium,* and *Rickettsia*, in most of the field‐collected populations (cf. Table 3). Using a multiplex PCR method developed by Zélé, Weill, and Magalhães (2018, detailed in Appendix S1), we found *Wolbachia* in 6 out of the 14 populations screened, whereas the remaining populations were free of symbionts (Table 3). Subsequently, to avoid incompatibilities among populations due to the presence of endosymbionts, a subset (*N* > 300 females) of each population selected to create the outbred populations (see below) was cured from endosymbiont infection by heat shock (continuous exposure to 33°C) for 6 generations, a method previously used in *T. urticae* for the same purpose (van Opijnen & Breeuwer, 1999). Due to potential side effects of the heat shock treatment, this procedure was used for all selected populations, independently of whether they were initially infected by symbionts. All populations were retested after the heat shock treatment to confirm the absence of symbionts.

**TABLE 3:** Infection by endosymbionts and ITS type

Species	Population	Symbionts	ITS type
*Tetranychus urticae*	HFM	Uninfected	n.a.
	MON	*Wolbachia*	
	ALP	Uninfected	
	DEF	Uninfected	
*Tetranychus evansi*	CG	Uninfected	T2
	ER	Uninfected	T2
	PBS	Uninfected	T1
	QG	*Wolbachia*	T1
	VC	*Wolbachia*	T2
	VIT	Uninfected	T1
	6M1	Uninfected	T1
*Tetranychus cinnabarinus*	6M2	Uninfected	n.a.
	LIM	*Wolbachia*	
	LNEC	*Wolbachia*	

Note

Spider mites of each population were tested in a pool (*N* = 50–100) for the presence of endosymbionts (*Wolbachia*, *Cardinium,* and *Rickettsia)*. In *Tetranychus evansi*, each population was characterized for its ITS type (T1 or T2).

Abbreviation: n.a., nonapplicable.

Reproductive incompatibilities due to genetic differentiation among populations of the same species are a common feature in many organisms (Corbett‐Detig, Zhou, Clark, Hartl, & Ayroles, 2013; Harrison & Larson, 2014; Jennings, Mazzi, Ritchie, & Hoikkala, 2011; Scopece, Lexer, Widmer, & Cozzolino, 2010), including spider mites (e.g., de Boer, 1982; Gotoh & Tokioka, 1996; Knegt et al., 2017; Sugasawa, Kitashima, & Gotoh, 2002). In our case study, to avoid well‐known reproductive incompatibilities between the green and red forms of *T. urticae* (e.g., de Boer, 1982; Gotoh & Tokioka, 1996; Sugasawa et al., 2002), we used populations of the green form only and discarded most populations of the red form (or *T. cinnabarinus*) after genetic identification. Additionally, in *T. evansi*, two highly incompatible major clades, I and II, have been identified based on the cytochrome oxidase complex I (COI) haplotypes and the internal transcribed spacer region (ITS; Boubou, Migeon, Roderick, & Navajas, 2011; Knegt et al., 2017). To avoid such incompatibility, we sequenced the ITS of *T. evansi* populations (Table 3; detailed in Appendix S1) and used only the populations with ITS type T1, corresponding to clade I, to create the outbred population.

## 4 CREATION OF OUTBRED POPULATIONS IN HAPLODIPLOIDS

**FIGURE 2:**
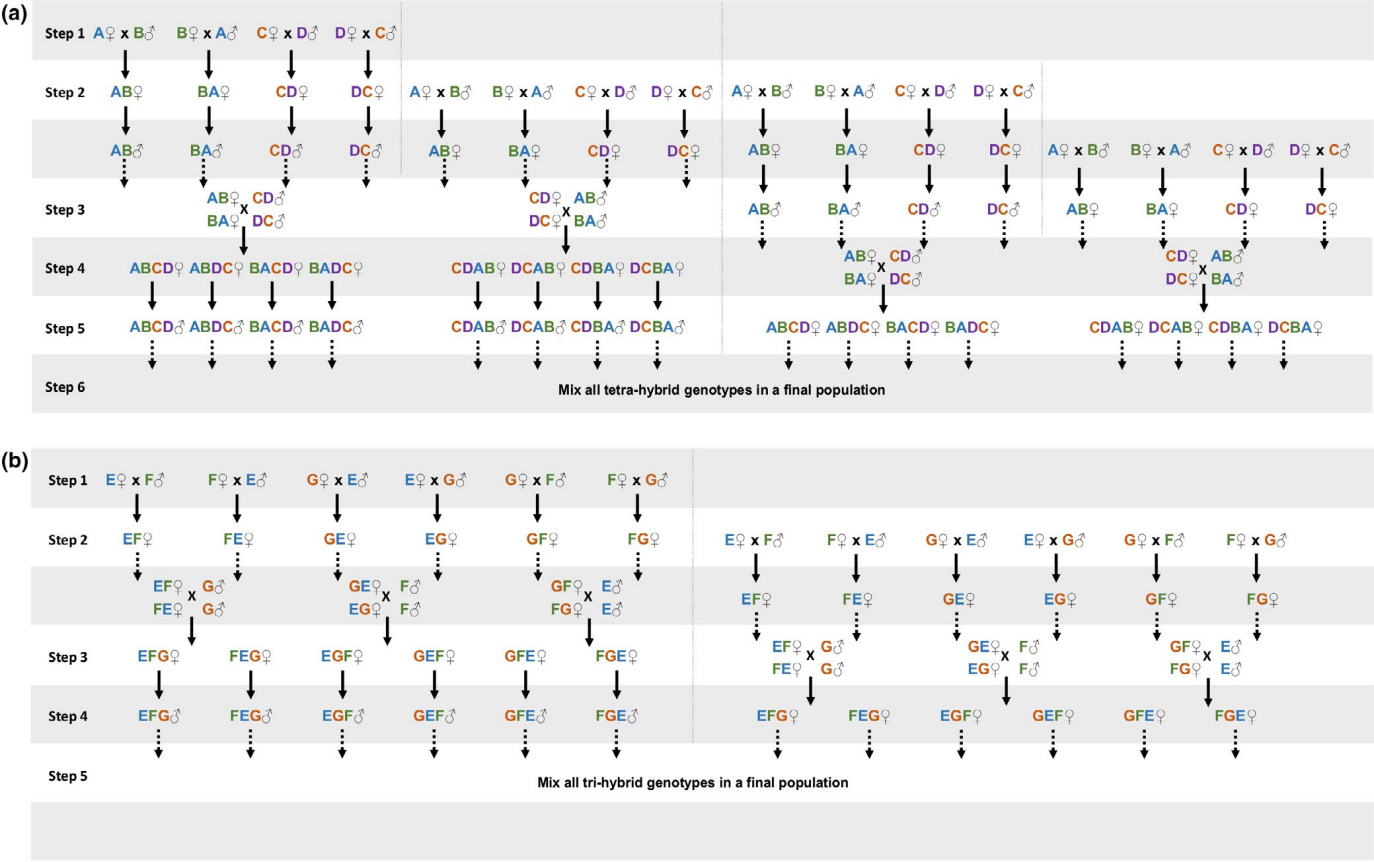
Creation of outbred populations on haplodiploids. Outbred populations of haplodiploid spider mites were created by performing controlled crosses between four (a) and three (b) populations collected in different locations. Letters represent the population from which the individuals stemmed (see Table 1). Each step represents the production of offspring to use in the crosses for the following generation: females were obtained from crosses between different genotypes and males from virgin females of a given genotype. Bold arrows represent the development of the offspring forming the next generation, and dashed arrows represent the use of hybrids for the subsequent crosses within a generation

Using different field‐collected populations to create outbred laboratory populations allows including genetic variation from different geographic locations, which increases the chances of capturing high variability. However, the number of populations used is limited by the logistical feasibility of protocols involving controlled crosses between those populations. Moreover, the complexity of such designs increases in haplodiploid systems (c.f. below) as compared to diploid systems. Here, to create outbred populations with high levels of standing genetic variation, we used 4 symbiont‐free à priori compatible populations of each species (the green form for *T. urticae* and Clade I for *T. evansi*). These populations were 6M1, PBS, QG, and VIT for *T. evansi* and ALP, DEF, MON, and HFM for *T. urticae* (Table 2; hereafter labeled A to D and E to H, respectively). The populations were merged by performing interpopulation crosses in a controlled match design, to avoid overrepresentation of genotypes from a given population (Figure 2). To this aim, 200 females from population A were crossed with 200 males from population B and vice versa; while 200 females from population C were individually crossed with 200 males from population D and vice versa. In this way, we obtained a hybrid F1 (AB and BA; CD and DC). However, because spider mites are haplodiploid with an arrhenotokous genetic system (Helle & Sabelis, 1985), only the F_1_ female offspring resulting from these crosses are hybrids. This characteristic adds one layer of complexity to controlled crossing design, as compared to diploid species. To form hybrid males, virgin hybrid F_1_ females were collected during their last molt, allowed to emerge as adult female and to lay unfertilized eggs for 48 hr. Subsequently, their offspring (haploid hybrid males) developed until adulthood. To synchronize the generations at which hybrid females and males were produced, a new generation of F_1_ hybrid females (again AB, BA, CD, and DC) was obtained by repeating the previous set of matings one generation later. These hybrid females were then crossed with hybrid males to produce a fully hybrid F_2_ (AB and BA hybrid females were crossed with CD or DC hybrid males and vice versa). Again, because males stem from unfertilized eggs, only the female offspring resulting from these crosses was a fully hybrid combination of the 4 populations (e.g., ABCD). These fully hybrid females were also isolated as virgin and their sons allowed to develop until adulthood. To synchronize the production of fully hybrid adult males and females, another cross of AB and BA females and CD or DC males (and vice versa) was performed simultaneously (Figure 2a). Finally, individuals of both genders of each of the 8 fully hybrid combinations performed (ABCD, ABDC, BACD, BADC, CDAB, CDBA, DCAB, and DCBA) were mixed to form the outbred population (Figure 2a).

This procedure was done to ensure an equal genetic representation (nuclear and mitochondrial) of each field population in the resulting outbred population. The number of crosses of each type (200) was chosen to ensure that the outbred population was founded by a sufficient number of genotypes for it to be representative of a natural population (>70 mated females; Sousa et al., 2019). Because some crosses did not produce viable offspring of at least one sex, we opted to found the outbred population with an equal number of individuals from each different combination (ex. ABCD), corresponding to the minimum number of genotypes obtained in those combinations. This means that the *T. evansi* outbred was founded with 72 females and 72 males from each of the 8 combinations performed (a total of 576 genotypes of each sex from 4 different locations).

During the creation of the *T. urticae* outbred population, hybrid breakdown was detected between the population HFM and the three others (i.e., ca. 75% of F_2_ offspring were inviable). The protocol was thus adapted to merge 3 populations instead of 4 (Figure 2b), by using a round‐robin design in which females/males of population E mated with males/females from population F or population G. These crosses produced hybrid females that would mate with males from the population not included in the parental crossing. The outbred population was founded with 51 females from each of 6 different combinations, corresponding to a total of 306 females. Because the total number of males was low (*N* = 197), we opted to use them all, even though the number across genotypes was not even.

## 5 CREATION OF INBRED LINES IN HAPLODIPLOIDS VIA FULL SIB‐MATING

A panel of inbred lines is an important biological tool as it allows determining the broad‐sense heritability of any trait and assessing the genetic correlations among traits. Inbred lines can also be used to test how different degrees of variation among traits affect ecological processes. For the knowledge provided by such tool to be more robust, it is important to maximize the coefficient of inbreeding of each line when designing their creation and to derive lines from the same population, as a way of partitioning its genetic variation across the lines. Below we describe a method to create inbred lines in haplodiploids through 15 generations of sib‐mating, as well as an estimation of their coefficient of inbreeding and the probability of obtaining a fully inbred line.

Inbred lines were initiated by isolating a mated female randomly sampled from the outbred population. Given full first‐male sperm precedence in spider mites, all descendants of a female stem from the same father (Rodrigues, Figueiredo, van Leeuwen, Olivieri, & Magalhães, 2020), which reduces genetic variance in the offspring as compared to species with mixed paternity. This protocol can easily be adapted to species with mixed paternity by initially isolating virgin males and females. In haplodiploids, a maximum of three different alleles (e.g., *x*, *y*, and *z*) can be initially sampled at each locus, independently of the number of alleles available for that locus in a population. Hence, 4 different types of mated females can be sampled, which correspond to 4 possible types of crosses: (*A*) a heterozygous female mated with a male that does not share any allele with her (e.g., [*xy*] * [*z*]); (*B*) a heterozygous female mated with a male that shares one of her alleles (e.g., [*xy*] * [*x*]); (*C*) a homozygous female mated with a male that does not share an allele with her (e.g., [*xx*] * [*y*]); or (*D*) a homozygous female mated with a male that has the same allele as her for that locus (e.g., [*xx*] * [*x*]).

Because we do not have access to the genotype of the mated female that initiated each line, we assume the most heterozygotic situation, that is, that we collected a [*xy*] female mated with a [*z*] male. By doing so, we conservatively underestimate the coefficient of inbreeding. This cross (type *A*) will produce ½ [*xz*] + ½ [*yz*] females and ½ [*x*] + ½ [*y*] males in the F_1_. If these daughters and sons mate randomly among themselves, crosses among sibs will occur with the following probability: ¼ [*xz*] * [*x*] (type *B*) + ¼ [*xz*] * [*y*] (type *A*) + ¼ [*yz*] * [*x*] (type *A*) + ¼ [*yz*] * [*y*] (type *B*). Thus, at each time step, type *A* crosses will result in sons and daughters that, if mated randomly among themselves, will produce ½ type *A* crosses and ½ type *B* crosses. Following the same reasoning, type *B* crosses, for example, [*xy*] * [*x*], will produce ½ [*xx*] + ½ [*xy*] females and ½ [*x*] + ½ [*y*] males. If these daughters and sons mate randomly, crosses among siblings will occur with the following probability: ¼ [*xx*] * [*x*] (type *D*) + ¼ [*xx*] * [*y*] (type *C*) + ¼ [*xy*] * [*x*] (type *B*) + ¼ [*xy*] * [*y*] (type *B*). Thus, type *B* crosses will result in sons and daughters that, if mated randomly among themselves, will produce ½ type *B* crosses, ¼ type *C* crosses, and ¼ type *D* crosses. From a type *C* cross, for example, [*xx*] * [*y*], only [*xy*] females, and [*x*] males will be produced, and thus, only [*xy*] * [*x*] crosses (type *B*) will occur. Finally, type *D* crosses, for example, [*xx*] * [*x*], will only produce [*xx*] females and [*x*] males, and thus, only type *D* crosses will occur.

Therefore, the frequencies of each type of cross, at each generation (*t* + 1), are given by the following equations:

$$A_{t+1}=\frac{A_{t}}{2}$$

$$B_{t+1}=\frac{A_{t}}{2}+\frac{B_{t}}{2}+C_{t}$$

$$C_{t+1}=\frac{B_{t}}{4}$$

$$D_{t+1}=\frac{B_{t}}{4}+D_{t}$$

The coefficient of inbreeding ( $f_{t}$ ), which corresponds to the probability that two alleles at one locus are identical by descent (Wright, 1921), is subsequently given by the following equation:

$$f_{t}=C_{t}+D_{t}$$

Alternatively, for full sib‐mating in haplodiploids, this coefficient can also be obtained directly as:

$$f_{t}=\frac{1}{4}+\frac{1}{4}f_{t-2}+\frac{1}{2}f_{t-1}$$

where the first two terms correspond to the probability of both alleles coming from the grandmother, being the alleles equal (first term), so that $f_{t}$ = 1, or different (second term), so that $f_{t}$ is equal to that of the grandmother $f_{t-2}$ , and the third term corresponds to the probability of one allele coming from the grandmother and the other from the grandfather, so that $f_{t}$ is the same as that of the mother $f_{t-1}$ .

**FIGURE 3:**
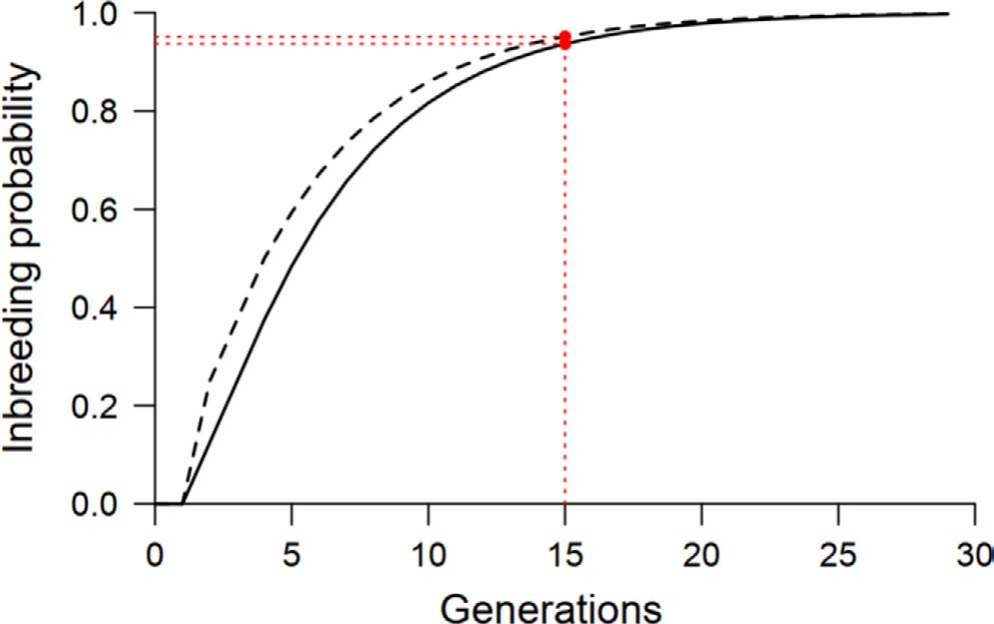
Inbreeding estimates from full sib‐mating crosses in haplodiploids. Coefficient of inbreeding (*f_t_*; dashed line) and probability of having a fully inbred line (*D_t_*; full line) for each discrete generation of full sib‐mating, starting from the most heterozygotic combination at one locus (e.g., a [*xy*] female mated with a [*z*] male). After 15 generations of sib‐mating, inbred lines of haplodiploids have a coefficient of inbreeding of 95.1% and a probability of being fully inbred of 93.6% (red lines and dots)

Both methods yield the same result, and assuming that generation 0 starts with a [*xy*] female mated with a [*z*] male (i.e., $A_{0}=1$ with the first method and $f_{1}$ = $f_{2}$ = 0 with the second method), we obtain a coefficient of inbreeding of 95.1% after 15 generations (Figure 3). However, the first method also allows estimating the probability of having a fully inbred line, which is given by the frequency of individuals stemming from fully homozygous crosses ( $D_{t}$ ). Again, assuming the most heterozygotic scenario, we obtain a probability of having a fully inbred line of 93.6% after 15 generations (Figure 3).

**FIGURE 4:**
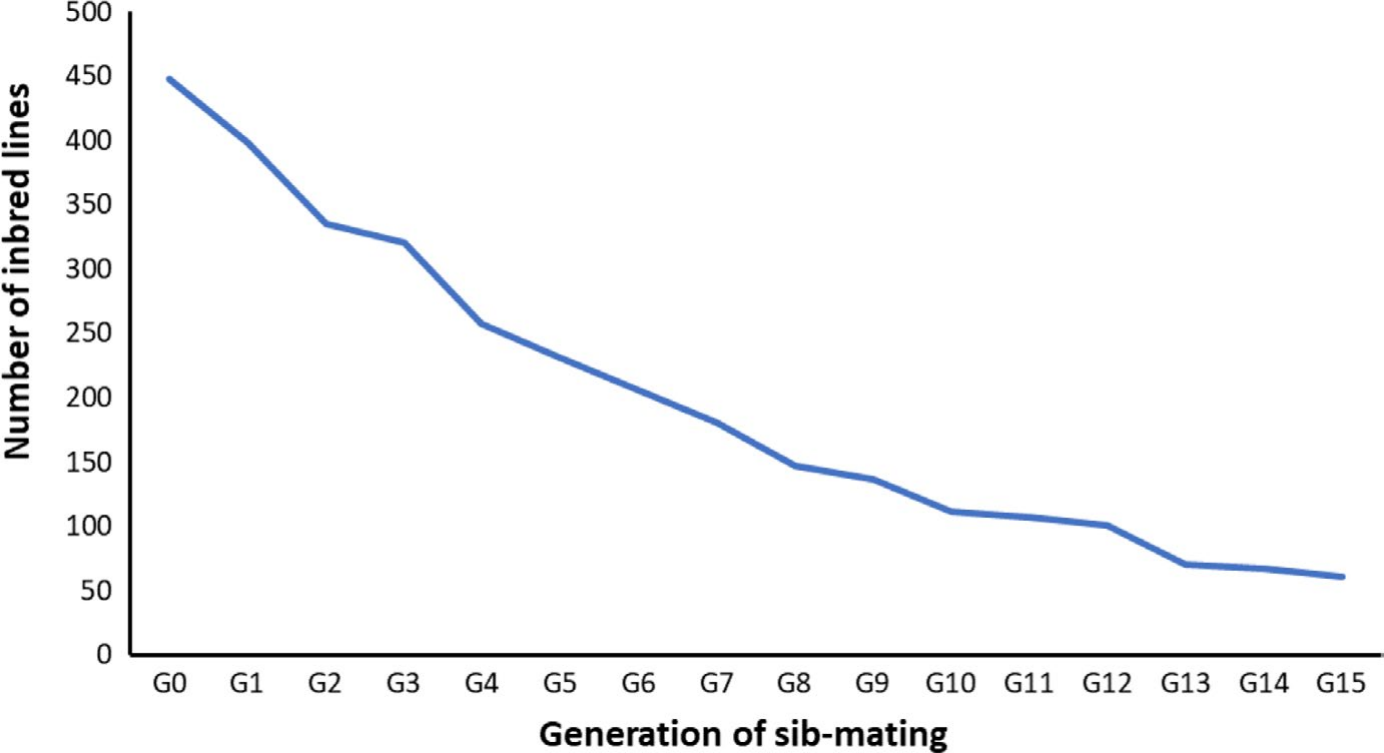
Loss of inbred lines during their creation. Number of inbred lines of *Tetranychus evansi* across 15 generations of sib‐mating

To create inbred female lines from the *T. evansi* outbred population*,* we randomly sampled 450 mated females, 2 generations after the creation of the population. These females were installed individually on leaf patches, where they laid eggs for 48 hr. The offspring of each female was then allowed to develop until adulthood (10–12 days) and to mate on that patch (i.e., sib‐matings). After 14 days, 3 mated females from each patch were isolated on 3 new patches and the same procedure was repeated. On the following generation, 3 sib‐mated females from one of the three patches only were isolated on 3 new patches and allowed to oviposit for 48 hr. The entire procedure was then repeated for 15 discrete generations. Having 3 replicates per line decreases the chances that lines are lost at each generation. However, despite this, many lines were lost due to the death of the female, null fecundity, no egg hatching, or no female or male offspring produced by a given female (Figure 4). After 15 generations of sib‐mating, each of the remaining inbred lines (*N* = 59) was transferred to individual patches of tomato plants kept on water‐soaked cotton in petri dishes and maintained in small numbers thereafter.

## 6 EXPERIMENTAL EVOLUTION PROTOCOL

**FIGURE 5:**
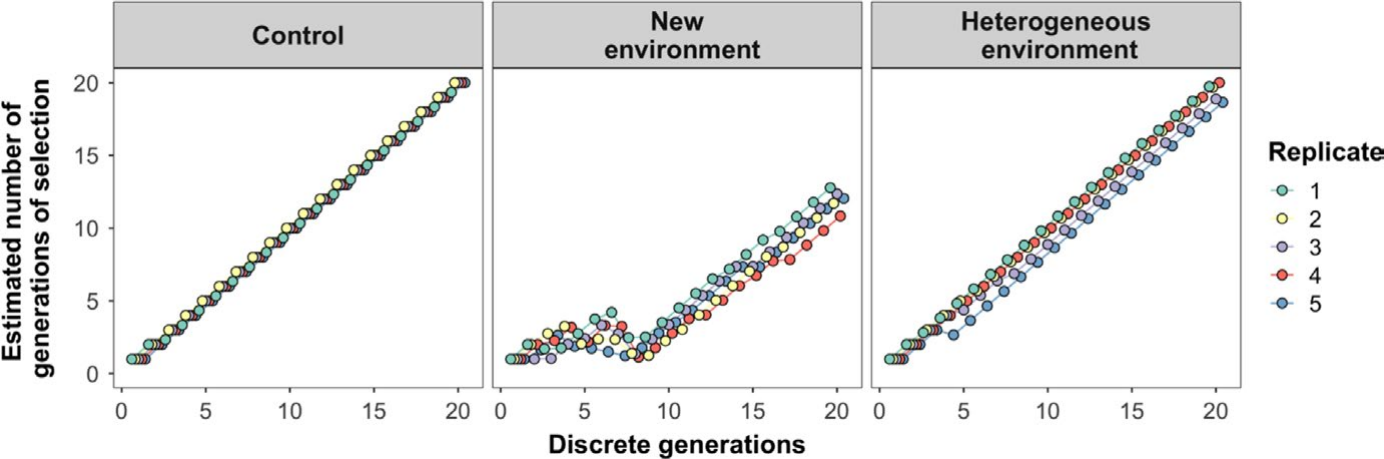
Estimated number of effective generations of selection during experimental evolution of spider mite populations exposed to different environments. Populations were exposed to an environment similar to that of the ancestral population (control), to a new environment, or to a mixture of both (heterogeneous environment). Because individuals from the *t *− 1 and base populations were often added in the selection regime corresponding to a new environment, the estimated number of generations decreases considerably relative to the other selection regimes. However, this procedure allowed populations to overcome the initial reduction in population size and to subsequently adapt to the selection regime imposed

Experimental evolution not only is a powerful method to detect adaptation to specific controlled factors but can also be combined with next‐generation sequencing techniques in order to identify and quantify individual loci contributing to adaptation (Magalhães & Matos, 2012; Schlötterer, Kofler, Versace, Tobler, & Franssen, 2015). For this purpose, several parameters of the experimental design, such as the number of founders, the number of generations, and the number of replicates per selection regime, must be carefully considered according to the system involved (Kofler & Schlötterer, 2014). However, during the course of the experiment these parameters may be affected due to predictable and unpredictable events (e.g., the loss of replicates due to the extinction of an experimental population). Here, we present a protocol that helps maximizing the prevention of such events. This experimental evolution protocol consists in transferring 220 randomly collected females from the outbred population to a box corresponding to a given selection regime. This number ensures >200 living (due to mortality in the transfer <5%) females founding each experimental population, which is the number needed to maximize the probability of detecting and quantifying responses to selection in spider mites (Sousa et al., 2019). This procedure is repeated 5 times per selection regime, as the replicate unit in experimental evolution studies is the population (Kawecki et al., 2012). All populations are maintained under the same environmental conditions except for variables that corresponded to each selection regime. In each generation, 220 randomly selected mated females are transferred to a new box with the same characteristics. The remaining individuals are maintained in the original box until the next transfer, creating a backup population (*t *− 1) for each replicate of each selective regime. Thus, if the 220 females cannot be found in a given box at the time of transfer, the remaining number is transferred from the backup population. If the sum of females found in the experimental population and its respective *t *− 1 backup does not add up to 220, the remaining number of females should be transferred from the base outbred population. This procedure allows maintaining the same population size for each replicate, preventing bottlenecks, and diminishing the chances of losing those populations. However, because every generation each experimental population may receive a different number of migrants from the *t *− 1 backup population and/or the base population (i.e., a different number of individuals are exposed to a different number of generations of selection), the average number of generations of selection of each replicate population might differ. The effective number of generations of selection can be estimated for each replicate population using the following equation:

$$Gen_{t+1}=1+\frac{N_{t}*Gen_{t}+N_{t-1}*Gen_{t-1}+N_{0}*Gen_{0}}{N_{\mathrm{total}}}$$

where $Gen_{t+1}$ corresponds to the effective number of generations of selection underwent by the individuals at the next generation. $Gen_{t}$ , $Gen_{t-1,}$ and $Gen_{0}$ correspond, respectively, to the effective number of generations of selection underwent by the current generation, the previous generation, and the base populations (i.e., not adapted to the new environment, so $Gen_{0}$ = 0). $N_{t}$ , $N_{t-1,}$ and $N_{0}$ correspond to the number of individuals transferred from the current generation of selection, the backup *t *− 1 box, and the base population, respectively, and $N_{\mathrm{total}}$ to the total number of adult females transferred. We provide an example of this formula by applying it to our system in Figure 5.

## 7 DISCUSSION

We describe the creation of biological resources (outbred and inbred populations) that maximize the maintenance of standing genetic variation in laboratory populations and, thus, increase the representativity of the responses described in laboratory conditions. As a case study, we present the creation of outbred populations for two spider mite species, *T. urticae* and *T. evansi*, by performing controlled crosses between recently collected field populations. In addition, we report a procedure to calculate the inbreeding coefficient, but also the probability of having a full inbred line when performing full sib‐mating in haplodiploids and apply it to the creation of inbred lines of *T. evansi*. Finally, we provide an outline of an experimental evolution protocol allowing the maintenance of constant densities across generations of selection, thereby reducing the risk of bottlenecks. Even though the methods we described are applied to haplodiploid species, they can easily be adapted to diploid systems as well. Thus, these protocols may be used in any species that can easily be sampled, have a relatively short generation time and can be maintained in the laboratory conditions in high numbers.

Undoubtedly the method we present here is time and work consuming. However, we believe that the advantages of creating such powerful tools, as we describe here, compensate the effort in the long run. The creation of outbred populations through controlled crosses allows to (a) incorporate genetic variability from different geographic collections, (b) detect reproductive incompatibilities between different genotypes and populations, and (c) incorporate an equal representativity of each population merged. All of these characteristics maximize the chances of maintaining a high amount of standing genetic variation in the outbred populations. By deriving the inbred lines from an outbred population, one also increases the chance of keeping that diversity among the lines. Additionally, the time spent with the creation of outbred populations gives the populations time to adapt to the laboratory conditions, a requirement before testing adaptive responses to other environments (Fragata et al., 2014; Matos, Avelar, & Rose, 2002; Simões et al., 2008). Moreover, while developing these tools, the populations collected may be thoroughly characterized, providing a preview of the variability expected in the derived outbred population and inbred lines.

Here, to illustrate the application of these methods, populations were collected from nearby locations. Therefore, the resulting populations do not encompass large areas of potential geographic variation, unlike, for example, the populations used to create the DSPR and the GDL panels, where the founding genotypes have distant geographic origins (Grenier et al., 2015; King et al., 2012). This may limit the standing genetic variation available, because of similar environmental conditions, and/or migration among populations. However, this is not very likely in the case of spider mites, as (a) experimental evolution studies performed with populations from a single location have repeatedly shown responses to selection (reviewed in Sousa et al., 2019), and (b) populations of spider mites show high genetic differentiation even within small geographic scales (e.g., Bailly, Migeon, & Navajas, 2004; Carbonnelle et al., 2007). Additionally, by founding the outbred populations with more than 500 individuals, the chances of obtaining large amounts of standing genetic variation are high.

Performing controlled crosses among field populations maximizes the chances of obtaining a highly outbred laboratory population. Indeed, this method allows controlling for assortative mating and for differences in fitness and/or mating competitive ability between genotypes. Therefore, it ensures an equal genetic representation of the nuclear and mitochondrial genomes of each population by equalizing the number of genotypes from each field population that will be incorporated in the outbred population. Finally, it allows the detection of reproductive incompatibilities between populations/genotypes, and the consequent exclusion of inviable crosses. Indeed, throughout the process of creation of these outbred populations, several crosses resulted in infertile females, or inviable offspring, indicating possible incompatibilities. Specifically, we detected hybrid breakdown (i.e., postzygotic reproductive isolation where F_2_ hybrids are inviable or sterile) between one population of *T. urticae* (HFM) and the others. None of these potential causes of reduction of the genetic variability in the final population would have been detected and prevented if the outbred population had been founded by mixing several individuals of different populations without controlling for the outcome of such crosses at the individual level, as previously done in other studies (Fricke & Arnqvist, 2007; Tucic et al., 1995).

Using outbred populations increases the chance that the responses observed are representative of the study species, which is a common shortcoming of laboratory studies. For example, Vala, Egas, Breeuwer, and Sabelis (2004) found that *T. urticae* females that are not infected with *Wolbachia* prefer uninfected over infected males, thereby potentially reducing the costs of incompatible mattings. However, this result was based on a single line, whereas a later study using an outbred population stemming from several field populations does not recapitulate this result (Rodrigues, Zélé, Santos, & Magalhães, 2018). Another example concerns the interaction between *T. urticae* and tomato plant defences. Although this herbivore generally induces plant defences, some field‐collected lines were shown to suppress them instead (Kant, Sabelis, Haring, & Schuurink, 2008). Therefore, capturing and maintaining natural variation in laboratory studies is highly relevant for understanding the ecology and evolution of the interaction between study organisms, such as spider mites, and many environmental factors, such as symbionts or plants. A particular example of studies that may profit from using outbred populations is those using experimental evolution. As genetic variance is the raw material for selection to act upon, having a highly outbred population to initiate experimental evolution will increase the chances of observing fast responses to selection. However, many experimental evolution studies have been performed with populations or strains collected from a single location, and in some cases from a small number of individuals (but see Fricke & Arnqvist, 2007; Zbinden et al., 2008). Spider mites are no exception to such contingencies (reviewed in Sousa et al., 2019). Therefore, the responses obtained may be idiosyncratic of the genetic background used. Providing the community with highly outbred laboratory populations may be very useful to test the generality of the responses reported and to perform future studies on other topics with a larger representation of the genetic variation of the species.

Moreover, in experimental evolution studies the initial diversity harbored by the ancestral populations may be quickly lost due to selection and/or stochastic events, leading to the extinction of experimental populations. Indeed, in environments that impose a strong selection pressure there is a high probability that the populations adapting to those conditions crash in a few generations. Additionally, unpredictable logistical problems that lead to the loss of experimental populations may occur. Here, we outline an experimental evolution protocol that allows using populations from the previous generation of selection (backup *t *− 1 populations) to ensure that the total population size remains constant across generations, thereby allowing populations to overcome the initial reduction in population size. In this way, it is possible to avoid losing replicates, as commonly occurs in experimental evolution studies (Cooper & Lenski, 2010; Schlötterer et al., 2015; Simões, Rose, Duarte, Gonçalves, & Matos, 2007). Such populations can thus be rescued and subsequently adapt to the selection regime imposed (Figure 5). In this case, it is important to calculate the effective number of generations of selection that populations have undergone, such as to correctly compare responses among selection regimes. Note that these *t *− 1 backup populations can be kept under relaxed conditions, thereby minimizing the workload necessary to maintain them.

Clearly, performing laboratory studies with outbred populations adds to their robustness. However, to characterize the different responses found in such outbred populations due to genetic variance, it is necessary to fix this variance along a panel of inbred lines, such that each genotype can be studied independently. For this purpose, one can derive a panel of inbred lines from an outbred population source, as done in *Drosophila melagonaster* (King et al., 2012; Mackay et al., 2012). The main advantage of this method is that the high genetic variability of the outbred population can be maintained among the inbred lines, while keeping the same genetic background. In particular, studying these lines allows a clear understanding of the phenotypic and genotypic variability for traits that may be relevant in many different contexts. Importantly, inbred lines can also be used to assess genetic correlations and trade‐offs between different traits (e.g., Everman et al., 2019; Howick & Lazzaro, 2017; Lafuente et al., 2018; Travers et al., 2015; Wang et al., 2017), including those measured in different environments (Howick & Lazzaro, 2014; Ørsted, Rohde, Hoffmann, Sørensen, & Kristensen, 2018; Unckless, Rottschaefer, & Lazzaro, 2015). Indeed, because all individuals of a given inbred line represent roughly the same genotype, responses of each genotype can be measured in different contexts. Additionally, such inbred lines can be used as a fixed genetic background against which the response of another population is studied. This may be particularly useful in the context of the evolution of biological interactions. For example, unraveling the evolution of sexual conflicts can be done by exposing individuals of the evolving sex to inbred lines of the nonevolving sex (e.g., Macke, Olivieri, & Magalhães, 2014). Also, the magnitude of G × G (genotype by genotype) in host parasite interactions has been addressed by exposing different lines of hosts and/or parasites to each other (Carpenter, Hadfield, Bangham, & Jiggins, 2012; Hudson, Fleming‐Davies, Páez, & Dwyer, 2016; Lambrechts, Fellous, & Koella, 2006; de Roode & Altizer, 2010). Furthermore, there is a recent increasing interest on how interindividual variation affects several ecological characteristics, such as species persistence and coexistence (Agashe, 2009; Bolnick et al., 2011; Forsman & Wennersten, 2016; Hart, Schreiber, & Levine, 2016; Lankau, 2009; Lichstein, Dushoff, Levin, & Pacala, 2007). Within this context, the creation of inbred lines may also be a useful tool. Finally, having the same background in the outbred population and the inbred lines allows comparing results stemming from both types of populations when tackling a common question.

The power of the biological resources described here, which can easily be adapted to other organisms, can be further potentiated if they are shared with collaborative laboratories and combined with increasingly fast advances on the genetic and genomic resources available. This will allow consistent and comparable studies that unquestionably will provide great advances in many different frameworks.

## CONFLICT OF INTEREST

None declared.

## AUTHOR CONTRIBUTIONS

**Diogo P. Godinho:** Investigation (lead); Methodology (equal); Visualization (equal); Writing‐original draft (equal). **Miguel A. Cruz:** Investigation (supporting); Writing‐review & editing (supporting). **Maud Charlery de la Masselière:** Investigation (supporting); Writing‐review & editing (supporting). **Jéssica T. Paulo:** Investigation (supporting); Writing‐review & editing (supporting). **Cátia Eira:** Investigation (supporting). **Inês Fragata:** Formal analysis (equal); Investigation (supporting); Visualization (equal); Writing‐review & editing (supporting). **Leonor R. Rodrigues:** Methodology (equal); Visualization (equal); Writing‐review & editing (lead). **Flore Zélé:** Formal analysis (equal); Methodology (equal); Visualization (equal); Writing‐review & editing (lead). **Sara Magalhães:** Conceptualization (lead); Funding acquisition (lead); Methodology (equal); Project administration (lead); Resources (lead); Supervision (lead); Writing‐original draft (equal).

## Supporting information

Appendix S1

## Data Availability

The dataset has been deposited in Figshare repository (https://doi.org/10.6084/m9.figshare.12263777).
