# Supplementary material for: Creating outbred and inbred populations in haplodiploids to measure adaptive responses in the laboratory
Source: Ecol Evol. 2020 Jul 7;10(14):7291–305. doi: 10.1002/ece3.6454 (PMC7391545; doi:10.1002/ece3.6454)
Supplement: Supplementary file 1 — Appendix S1 [file ECE3-10-7291-s001.docx]

**Appendix 1 Identification of *Tetranychus* species and detection of endosymbiont infection.**

1. **DNA extraction**

A pool of 50-100 female spider mites from each population was used to extract the DNA using the Sigma-Aldrich protocol (GenElute™ Mammalian Genomic DNA Miniprep Kit, Sigma-Aldrich, St. Louis, MO, USA). This DNA was then used for identification of spider mite species and *T. evansi* ITS type, and for the diagnostic of endosymbiont infection. Note that previous sensitivity tests showed that the multiplex PCRs used for species identification and symbiont infections allowed successful detection of target genes at very low density (up to 1%; i.e. 1 mite in a pool of 100; Zélé et al. 2018c).

1. **Species identification**

To identify each population of spider mites at the species level, we used the multiplex PCR developed by Zélé et al. (2018c). Briefly, we used a *Tetranychus*-generalist forward primer and different reverse primers specific to each target species (Table A1). The amplification conditions were the following: 15 min at 95 °C, followed by 35 cycles of 94 °C for 30 s, 58 °C for 1 min 30 s (annealing), 72 °C for 1 min and a final step at 72 °C for 10 min.

**Table A1** List of primers used in multiplex to identify *Tetranychus urticae, T. ludeni* and *T. evansi*

| **Species** | **Target gene** | **Primer name** | **Sequence (5´- 3´)** |
| --- | --- | --- | --- |
| *Tetranychus sp.* | 5.8S | TspG_F | TAATCGGTGCGAATTGCAGG |
| *T. urticae* | 28S | TuS_R | ATGTTTATTTGTGTTGTTTGCAAGC |
| *T. ludeni* | ITS2 | TlS_R | GAATGAAATAGATACTATTTGTGATTC |
| *T. evansi* | ITS2 | TeS_R | GATTCATGTATACAYATATAAATATATGC |

Identification of the ITS2 types of each population of *T. evansi* was subsequently performed by PCR amplification and sequencing of a fragment of the nuclear ribosomal DNA (rDNA) ITS2 region using the primers ITS2a (5’-TACCAATCGATGAAGAACGTAGC-3’) and ITS2b (5’ATATGCTTAAATTCAGGGGG-3’) developed by Hurtado et al. (2008). The conditions for amplification were the same than for the multiplex PCR, except for the annealing temperature that was 50ºC. The PCR products were sent to Stabvida (Caparica, Portugal) for purification and sequencing.

1. **Endosymbiont detection**

To detect infection by *Wolbachia*, *Cardinium* and/or *Rickettsia* in our populations, we used multiplex PCR developed by Zélé et al. (2018c). Briefly, we used forward and reverse primers for one specific gene of each endosymbiont, as well as spider mite generalist primers to distinguish uninfected individuals from PCR failure (Table A2). The amplification conditions were the same as for the species identification multiplex PCR, except for the annealing temperature that was 65 ºC.

**Table A2** List of primers used in multiplex to detect simultaneous infection by *Wolbachia*, *Cardinium* and / or *Rickettsia*

| **Target gene** | **Primer name** | **Sequence (5´- 3´)** |
| --- | --- | --- |
| *Tetranychus* ITS1 (partial) | ITS1G_F | AGGTGAACCTGCGGAAGGATCATTAACG |
|  | ITS1G_R | CCTTCTTTAAACCTTGCCGTCAGCATAAGC |
| *Wolbachia* wsp | WSPTG_F | GTTGGTGTTGGTGCAGCGTATGTAAGC |
|  | WSPTG_R | AGTGCTGTAAAGAACTTTGATTCCGCCATC |
| *Cardinium* 16S rRNA | CARDTG_F | GGCGGCTTATTAAGTCAGTTGTGAAATCCT |
|  | CARDTG_R | GCTGCCTACGCTATTGGTATTCCTTATGAT |
| *Rickettsia* gtlA | RICTG_F | AGGCTAATGGGCTTTGGTCATCGTGTAT |
|  | RICTG_R | TGTGCCATCCAGCCTACTGTTCTTGC |
